# Interhemispheric Interactions between the Human Primary Somatosensory Cortices

**DOI:** 10.1371/journal.pone.0016150

**Published:** 2011-02-10

**Authors:** Patrick Ragert, Till Nierhaus, Leonardo G. Cohen, Arno Villringer

**Affiliations:** 1 Department of Neurology, Max-Planck-Institute for Human Cognitive and Brain Sciences, Leipzig, Germany; 2 Human Cortical Physiology Section (HCPS), National Institute of Neurological Disorders and Stroke (NINDS), National Institutes of Health (NIH), Bethesda, Maryland, United States of America; 3 Berlin Neuroimaging Center (BNIC) and Department of Neurology, Charité, Berlin, Germany; 4 Berlin School of Mind and Brain, Humboldt University, Berlin, Germany; Claremont Colleges, United States of America

## Abstract

In the somatosensory domain it is still unclear at which processing stage information reaches the opposite hemispheres. Due to dense transcallosal connections, the secondary somatosensory cortex (S2) has been proposed to be the key candidate for interhemispheric information transfer. However, recent animal studies showed that the primary somatosensory cortex (S1) might as well account for interhemispheric information transfer. Using paired median nerve somatosensory evoked potential recordings in humans we tested the hypothesis that interhemispheric inhibitory interactions in the somatosensory system occur already in an early cortical processing stage such as S1. Conditioning right S1 by electrical median nerve (MN) stimulation of the left MN (CS) resulted in a significant reduction of the N20 response in the target (left) S1 relative to a test stimulus (TS) to the right MN alone when the interstimulus interval between CS and TS was between 20 and 25 ms. No such changes were observed for later cortical components such as the N20/P25, N30, P40 and N60 amplitude. Additionally, the subcortically generated P14 response in left S1 was also not affected. These results document the existence of interhemispheric inhibitory interactions between S1 in human subjects in the critical time interval of 20–25 ms after median nerve stimulation.

## Introduction

One of the basic principles in the organization of the human brain is that each cerebral hemisphere processes information from the opposite side of the body. Based on animal experiments, there is convincing evidence that callosal projections contribute to interhemispheric integration and transfer of information. That such projections can convey information between the hemispheres in human subjects is suggested by e.g. the detection of evoked potentials over primary motor cortex (M1) following electrical or magnetic stimulation of the contralateral M1 (for review see [1]). Maladaptive functioning of interhemispheric interactions such as alterations in interhemispheric inhibition (IHI) has been described in chronic stroke and is thought to be one of the key candidates for motor impairments in these patients [2]. For example, an abnormally high interhemispheric inhibitory drive from M1 of the intact to the lesioned hemisphere has been shown to be associated with poor motor performance [3].

Compared to the findings in the motor cortex, evidence for the existence of interhemispheric transfer in other modalities such as the somatosensory system still remains elusive. There is some evidence that interhemispheric information transfer may be an exclusive attribute of the secondary somatosensory cortex (S2), which receives extensive interhemispheric projections from the contralateral body part [4], [5]. Evidence for interhemispheric transfer of tactile information in human subjects comes from patients that underwent resection of the posterior half of the corpus callosum [6], [7]. These studies demonstrated that bilateral activation of S2 requires the integrity of the posterior body of the corpus callosum. Furthermore, it has been shown that the size of the intermediate callosal truncus contributes to the timing and amplitude of the ipsilateral S2 source activity [5]. The transcallosal conduction time between homologous S2 was estimated in previous studies and is supposed to range between 10–20 ms [5], [8].

Recent animal studies indicate that also parts of the primary somatosensory cortex (S1) such as area 2 have relatively dense callosal connections while areas 3b and 1 have only few connections. This in turn provides another potential substrate for interhemispheric transfer of tactile information (for review see [9], [10]). Therefore, it is reasonable to assume that normal interhemispheric transfer of tactile information might take place not only between S2 but also at an earlier sensory processing stage such as between S1 [11], [12], [13], [14], [15]. For example, Hlushchuk and colleagues (2006) found that unilateral touch of fingers is associated, apart from activation in contralateral S1, with a deactivation of the ipsilateral S1 [16]. They suggest that the observed ipsilateral S1 deactivation might result from transcallosal inhibition between both S1.

Assuming that interhemispheric information transfer in humans occurs already between S1, it remains to be determined which critical time window contribute to interhemispheric transfer of sensory information. Furthermore, it is still unclear whether interhemispheric communication in S1 relies predominately on inhibitory or excitatory interhemispheric interactions. Based on these considerations we hypothesized the existence of interhemispheric inhibitory interactions linking the two S1 in humans at an early stage of somatosensory processing.

## Materials and Methods

### Experimental procedures

#### Subjects

We studied twelve healthy volunteers between 22 and 32 years of age (26.8±2.9 years (SD); 4/12 females). They gave written informed consent to participate in the experiment according to the declaration of Helsinki and the ethics committee of Leipzig approved the study. Prior to participation, all volunteers underwent a comprehensive neurological examination and were without acute or chronic medication. According to the Oldfield questionnaire for the assessment of handedness [17], all subjects were right-handed (laterality score: +100±11 (median ± range) over a range of −100 (fully left-handed) and +100 (fully right-handed)).

### Main Experiment

**Figure 1:**
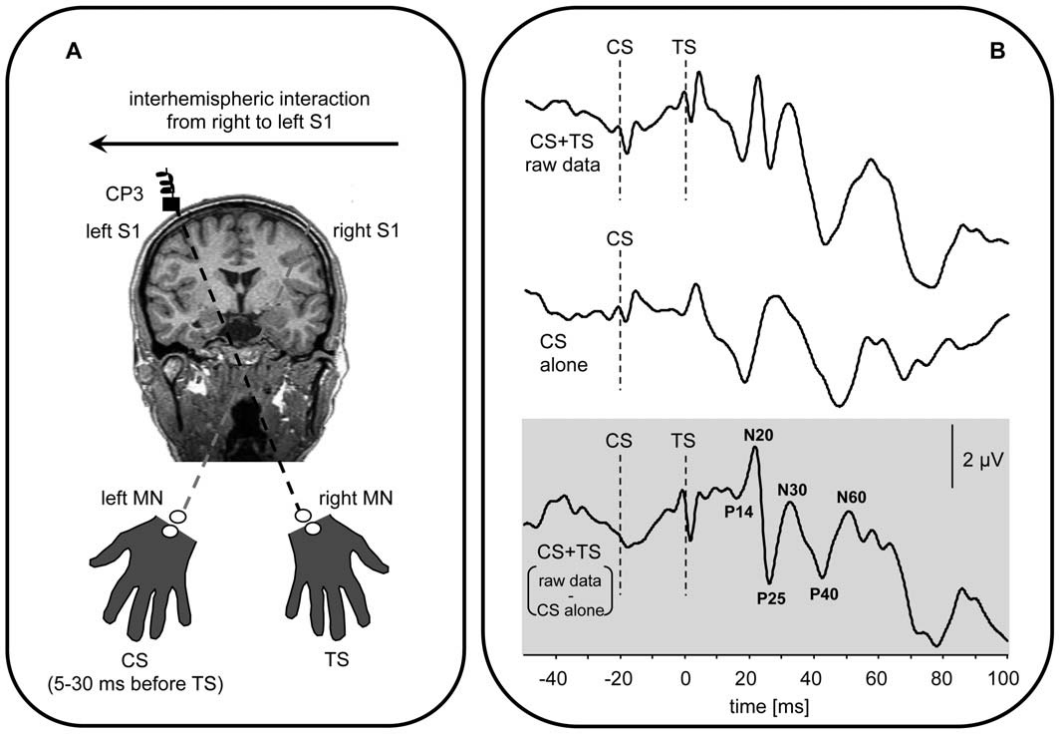
Experimental design of the paired median nerve somatosensory evoked potential recordings (PMNSEPs). (**A**) Interhemispheric interactions between homologous primary somatosensory cortices (S1) were studied using paired median nerve somatosensory evoked potential recordings (PMNSEPs). In the paired median nerve paradigm, suprathreshold peripheral stimulation of the left median nerve (MN) served as conditioning stimulus (CS) and always preceded right MN stimulation (test stimulus (TS)) by 5–30 ms while recording SEPs over the left (target) S1. Additionally, SEP responses to a CS (left MN) and TS (right MN) alone were recorded over left S1 (for details see text). Analysis of PMNSEPs was performed at electrode CP3. (**B**) Example traces of an individual subject illustrating the subtraction method used. In brief, the response of a left MN CS alone stimulation (middle trace) over left S1 was subtracted from the raw CS+TS response over left S1 (upper trace). Final analysis of the PMNSEP data was performed on the CS+TS SEPs (CS+TS raw data – CS alone, lower trace). For details see text. Amplitudes of interest (P14, N20, N20/P25, N30, P40, N60) are marked on the lower trace.

Interhemispheric interactions between homologous primary somatosensory cortices (S1) were studied using a novel paradigm consisting of paired median nerve somatosensory evoked potential recordings (PMNSEPs) at suprathreshold (1.60±0,79 V for left median nerve, 1,46±0,49 for right median nerve (mean ± stdev.)) intensities. In the paired median nerve paradigm, peripheral stimulation of the left median nerve (MN) served as conditioning stimulus (CS) and always preceded right MN stimulation (test stimulus (TS)) by 5–30 ms while recording somatosensory evoked potentials (SEPs) over the left (target) S1. Additionally, SEP responses to a CS (left MN) and TS (right MN) alone were recorded over left S1 (see Fig. 1). Using this design, we were able to study possible interhemispheric interactions from right to left S1. Changes in early SEP components in the PMNSEPs relative to TS alone would give information about interhemispheric facilitation or inhibition between right and left primary somatosensory cortices.

Using a visual analogue scale (VAS), healthy volunteers rated their attention level toward the task (range 1–10; 1 = no attention, 10 = high attention), their perception of fatigue (range 1–10; 1 = strong fatigue, 10 = no fatigue) as well as their discomfort (range 1–10; 1 = no discomfort, 10 = strong discomfort) twice during the experiment (before and after the PMNSEP recordings).

### Paired median nerve somatosensory evoked potential recordings (PMNSEPs)

SEPs were recorded after paired electrical median nerve stimulation of the right and left hand (PMNSEPs). Electrical pulses were generated and triggered using Spike2 software package (Version 5.04, Cambridge Electronic Design, Cambridge, UK) together with a CED Power 1401 interface (Cambridge Electronic Design Ltd., UK) and presented to the subjects using a DS5 isolated bipolar constant current stimulator (Digitimer Ltd, Welwyn Garden City, Hertfordshire, UK). For PMNSEP recordings, standard block-electrodes were placed to the right and left median nerve (MN) at the level of the wrist (cathode proximal). MN stimulation was performed using a pulse width of 100 µs and a repetition rate of 2 Hz. Electrical stimulation intensity was adjusted for the the left and right MN individually to produce a small but visible muscular twitch in the thumb (1.60±0,79 V for left MN, 1,46±0,49 for right MN (mean ± stdev.). The chosen stimulation intensity was not perceived as uncomfortable or painful by the subjects.

PMNSEP recordings were performed using a MR compatible electroencephalogram (EEG) system (Brainvision (UK) Ltd., BrainAmp MR plus) from 32 scalp positions evenly distributed over both hemispheres according to the International 10–20 system. During recordings, the midfrontal electrode (FPz) was used as reference and an electrode at the sternum served as ground electrode. The skin electrode impedance was always kept below 5 kΩ. PMNSEPs were acquired with a band-pass filter between 0.1 and 1000 Hz and digitized with a sampling rate of 5000 Hz (sampling interval 200 µs) in epochs from 100 ms before and 400 ms after the stimulus pairs.

During PMNSEPs, left MN stimulation always proceeded right MN stimulation using 6 different interstimulus intervals (ISIs, CS+TS) ranging from 5–30 ms in 5 ms steps (see Fig. 1A). The choice of ISIs was motivated by previous studies showing transcallosal conduction times in the somatosensory system (S2) ranging between 10–20 ms [8]. Since transcallosal conduction times between homologous S1 might slightly differ as compared to S2 we therefore tested a broader range of ISIs (5–30 ms). Additionally, a test stimulus (TS alone, right MN) as well as a control stimulus (CS alone, left MN) was applied while recording PMNSEPs over the left (target) hemisphere. The order of the conditions (CS+TS (5–30ms), TS and CS alone) was pseudo-randomized during the experiment. A total number of 1200 stimulus related epochs were recorded with 150 epochs for each condition (6 ISIs (CS+TS), TS alone and CS alone).

PMNSEPs were analyzed offline using a custom built program running under Matlab environment (Mathworks, Sherborn, MA, USA, Version 7.7). Epochs were digitally filtered using a standard 3^rd^ order band-pass Butterworth filter (1–200 Hz) and each condition was averaged. Analysis was performed on electrode CP3 over the left (target) hemisphere.

A potential problem using the PMNSEP technique is that after paired-pulse stimulation (CS+TS) the response to the second (test) stimulus (TS) might be influenced by an ipsilateral response component (rather than the transcallosal effect to be tested) of the first (conditioning) stimulus (CS). Therefore, the response of a left MN CS alone stimulation over left S1 was subtracted from the raw CS+TS response over left S1. We used the following procedure for subtraction: In a first step, the average SEP response at electrode CP3 (left S1) was calculated for each condition (6 ISIs (CS+TS raw data)) and for CS alone stimulation (left MN). Subsequently, the resulting SEP (epoch from 100 ms before and 400 ms after MN stimulation) for the CS alone stimulation was subtracted from each of the 6 CS+TS raw conditions (ISI 5–30 ms, see also Fig. 1B). Final analysis of the PMNSEP data was performed on the CS+TS SEPs (CS+TS raw data – CS alone).

For all subjects, the following SEP amplitudes with cortical origin were analyzed separately: N20, N20/P25 complex, N30, P40 and N60. The subcortical P14 component [18] was additionally assessed but could only be reliably identified in 8 out of 12 subjects. The N20 amplitude was assessed as the difference between the onset (around 14–16 ms) and the first negative peak usually ranging around 17–22 ms after stimulus onset (see also [19]). In case the P14 could be detected in some subjects, the N20 response was measured from the peak of the P14 to the peak of the N20. The amplitude of the N20/P25 complex was measured as the difference between the N20 peak and maximum subsequent positivity. The N30 amplitude was measured as the difference between the N20/P25 complex peak and maximum subsequent negativity, the P40 amplitude as the difference between the N30 peak and maximum subsequent positivity as well as the N60 amplitude as the difference between the P40 peak and maximum subsequent negativity. The subcortical P14 component was assessed, if possible, as the difference between the baseline and the first positive peak ranging around 12–17ms post stimulus onset.

### Statistical analysis

Data were analyzed using the PASW software package for Windows version 18. For statistical analyses, we first used two-way repeated measures ANOVA (ANOVA_RM_, if necessary corrected for non-sphericity) with factor AMPLITUDE (N20, N20/P25, N30, P40 and N60) and ISI (TS alone, 5, 10, 15, 20, 25, 30 ms). In a second step, we performed six one-way ANOVA_RM_ with factor ISI for all amplitudes tested. Subsequently, post-hoc tests (Bonferroni-corrected) were performed to identity differences in specific PMNSEP amplitudes of each ISI compared to TS alone. For post-hoc tests, the significance level was set to p = 0.008 to correct for multiple comparisons. All figures represent group data. Error bars refer to the standard error (s.e.m.) of the measurements.

## Results

There was no statistically significant change in our assessment of attention (pre: 8.25±0.51, post: 8.00±0.42; p>0.05; range 1–10; 1 = no attention, 10 = high attention), fatigue (pre: 7.87±0.48, post: 7.87±0.45; p>0.05; range 1–10; 1 = no fatigue, 10 = strong fatigue) or discomfort (pre: 1.00±0.00, post: 1.00±0.00; p>0.05; range 1–10; 1 = no discomfort, 10 = strong discomfort) before (pre) and after (post) the experiment. None of the subjects reported any discomfort during the paired median nerve somatosensory evoked potential (PMNSEP) recordings.

A two-way ANOVA_RM_ revealed a significant effect of AMPLITUDE (N20, N20/P25, N30, P40 and N60: F_(4,44)_ = 5.031; p = 0.031) and ISI (TS alone, 5–30 ms CS+TS: F_(6,66)_ = 3.746; p = 0.027) on PMNSEP.

**Figure 2:**
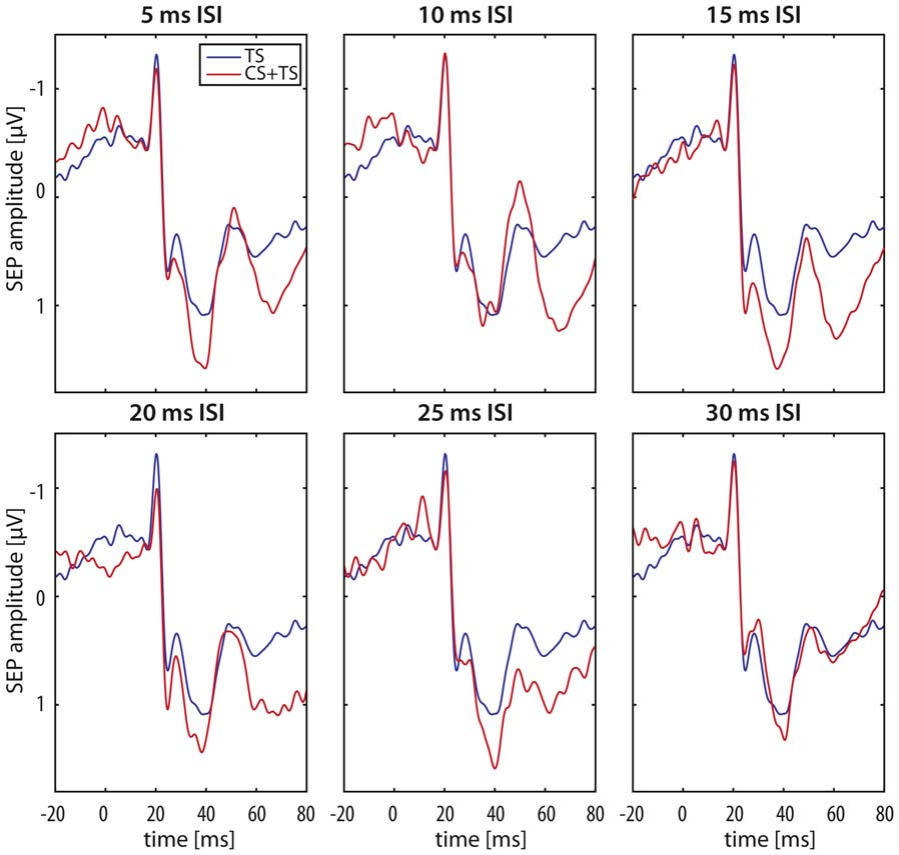
Example traces of a single subject PMNSEP response at electrode CP3 for all CS+TS conditions (5–30ms, red trace) relative to TS alone (blue trace). Please note that the PMNSEPs for all CS+TS conditions are superimposed (shifted) on the N20 onset of the TS alone condition for display purpose only. For average group data please see Table 1.

**Figure 3:**
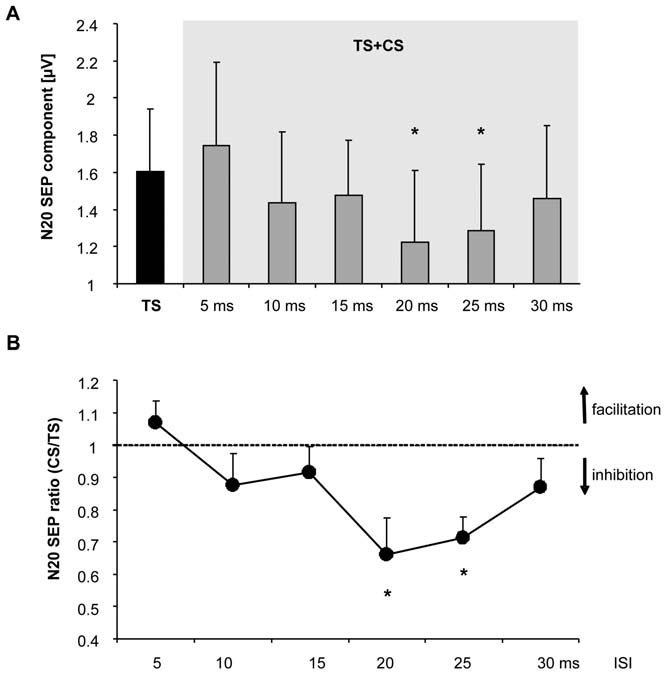
Effect of conditioning the right S1 on N20 response of the contralateral (left) S1. Conditioning the right S1 by electrical median nerve stimulation of the left MN (CS) resulted in a statistically significant reduction of the N20 response in the target (left) S1 relative to a test stimulus to the right MN (TS) alone when the interstimulus interval between CS and TS was between 20 and 25 ms. (A) Non-normalized N20 amplitudes for all conditions tested (Ts alone, 5, 10, 15, 20, 25 and 30 ms) (B) Normalized N20 amplitudes of the left S1 are displayed as a ratio between the CS+TS conditions (5–30 ms ISI) relative to TS alone (CS+TS/TS ratio). Asterisks represent significant differences relative to TS alone (significance level p<0.008, corrected for multiple comparisons).

Conditioning the right S1 by electrical median nerve stimulation of the left MN (CS) resulted in a significant reduction of the N20 response to right MN stimulation in the target (left) S1 (one-way ANOVA_RM_ with factor ISI (TS alone, 5–30ms): F_(6,66)_ = 3.951; p = 0.031, see Figures 2 and 3). No such changes could be observed for the other amplitudes tested (N20/P25: F_(6,66)_ = 2.380; p = 0.085; N30: F_(6,66)_ = 0.847; p = 0.458; P40: F_(6,66)_ = 0.920; p = 0.463; N60: F_(6,66)_ = 2.691; p = 0.066).

Post-hoc analysis revealed that the N20 response of the left S1 (relative to TS alone) was inhibited from 1.66±0.33 µV (TS alone) to 1.22±0.38 µV at a CS+TS ISI of 20 ms (CS+TS/TS ratio: 38.98±8.20%, paired T-Test: p = 0.0038). We also found an inhibition of the N20 response at a CS+TS ISI of 25 ms from 1.66±0.33 µV (TS alone) to 1.28±0.35 µV (CS+TS/TS ratio: 34.15±8.41%, paired T-Test: p = 0.0013; see Figures 2 and 3 and Table 1). No such changes on other PMNSEP components were observed for CS+TS ISIs of 5, 10, 15 and 30 ms (p>0.05, see Table 1).

**Table 1:** PMNSEP amplitudes [µV] for electrode CP3 of the left (target) S1 for unilateral (TS alone) and bilateral (5–30 ms) median nerve stimulation.

	ISI [µV]						
Amplitude	TS alone	5 ms	10 ms	15 ms	20 ms	25 ms	30 ms
**P15 (n = 8)**	0.17±0.05	0.28±0.06	0.25±0.06	0.33±0.06	0.25±0.06	0.39±0.11	0.29±0.10
**N20**	1.60±0.33	1.74±0.44	1.44±0.38	1.47±0.30	1.22±0.38**	1.29±0.36**	1.46±0.39
**N20/P25**	4.83±1.55	5.26±1.67	4.94±1.53	5.08±1.48	4.80±1.36	4.49±1.28	4.44±1.34
**N30**	2.36±1.11	2.86±1.41	2.60±1.33	2.66±1.37	2.55±1.12	2.76±1.22	2.37±1.11
**P40**	1.88±0.36	2.26±0.46	1.96±0.44	1.89±0.38	1.96±0.38	1.79±0.28	1.99±0.30
**N60**	2.63±0.57	3.62±0.76	2.99±0.65	3.01±0.67	2.57±0.51	2.89±0.50	2.95±0.46

Asterisks represent significant differences relative to TS alone (significance level p<0.008, corrected for multiple comparisons).

To investigate if the attenuated effect on the N20 component (CS+TS ISI 20 and 25 ms) of the left S1 occurred already at a subcortical level, amplitude changes of the subcortically generated P14 component of left S1 for all CS+TS conditions were identified and analyzed in 8 out of 12 subjects. We found that the P14 component did not change in response to the CS+TS ISIs relative to TS alone (one-way ANOVA_RM_ with factor ISI (TS, 5–30ms): F_(6,42)_ = 1.051; p>0.05, see Table 1).

## Discussion

Our results demonstrate that a conditioning stimulus reaching the right S1 attenuates the early cortical N20 response in the left S1 activity at interstimulus intervals of 20 and 25 ms, providing direct evidence for transcallosal information transfer at an early stage of cortical processing in the human somatosensory system. Previous work reported that transcallosal information transfer of propioceptive information from distal body parts can take place in the secondary somatosensory cortex (S2) [10], [20]. The bilateral activation of S2 following unilateral sensory stimulation has been related to the presence of dense transcallosal connections between both S2 [21].

Early tracer injection studies in animals indicated that callosal connections between the postcentral gyri exist for face and trunk areas [22]. More recently, it has been shown that homologous representations in the postcentral gyrus in Brodman areas (BA) 1, 2 and 3b are directly or indirectly connected via callosal fibers [10], [23]. This is in line with previous findings showing that callosally mediated ipsilateral potentials in the barrel cortex disappeared after applying a lesion to the contralateral sensory cortex [12], [24]. Furthermore, disruption of function in the postcentral gyrus (BA3) by cooling resulted in an augmentation of activity and enlargements of receptive fields of neurons in the homologous S1 suggesting that a potential role of callosal fibers in S1 is to mediate inhibitory information across hemispheres [11]. The latter finding is in line with our results in human subjects. The reduced N20 component seems best explained by an inhibitory drive from the right to the left S1 when the peripheral conditioning stimulus (CS) to the right S1 was delivered either 20 or 25 ms before the peripheral test stimulus (TS) to left S1. Since we did not find facilitation of SEP responses at any other interstimulus interval between CS and TS, transcallosal information transfer in early processing stages of the somatosensory system seems to be mainly inhibitory.

These findings are consistent with those of Werhahn et al. (2002) who found that anaesthesia of one hand (resulting in decreased input to the contralateral S1) resulted in a facilitation of the opposite S1, as tested with early cortical components of the somatosensory evoked potentials [13].

Functional MRI studies showed that unilateral stimulation of fingers is associated with a blood oxygenation level-dependent (BOLD) activation of contralateral S1 and S2 as well as a transient deactivation in BOLD signal of the ipsilateral S1 and of the primary motor cortex (M1) of both hemisphere [16]. While the neuronal mechanisms behind negative BOLD signals in humans are not well understood, animal data seems to indicate that it could reflect neuronal inhibition [25].

The present study documents the existence and temporal specificity of interhemispheric inhibitory influences between human primary somatosensory cortices. Since the (reduced) N20 SEP response reflects activity in layer 4, the input layer of BA3b [26], [27], the transcallosal inhibition can be assumed to take place at BA3b in a critical time window of 20–25 ms.

Given that the input layer of BA3b also receives thalamic inputs, it is important to consider that the reduction of the N20 response may be at least partially mediated by altered processing already in subcortical regions such as the ventroposterior parietal nucleus (VPL). Even though this possibility cannot be entirely ruled out by our study, the fact that the P14 response, originated in VPL [18], remained unchanged under all conditions tested rendering a subcortical origin of inhibition in S1 unlikely.

Another potential interpretation of the present data is that the inhibition of the N20 response in the left S1 by conditioning the homologous S1 is mediated through modulation of both M1. Since afferent inputs elicited by suprathreshold median nerve stimulation not only reach S1 but also approximately 4 ms later the ipsilateral M1 via direct corticocortical fibers [28] and somatosensory input from one hand influences the ipsilateral motor cortex in humans it is possible that the changes seen in the left S1 are a result of a modulation of interhemispheric communication across homologous M1, although this possibility is unlikely. Sensory information reaches the opposite S1 approx. 20 ms after MN stimulation. After another 5 ms, sensory information is assumed to reach the ipsilateral M1 [28]. From the ipsilateral M1 it will take another 6–50 ms to inhibit the opposite M1 (for review see [29]) which in turn will affect the S1 on the same hemisphere 5 ms later. Therefore it might take up to 36–80 ms for afferent inputs elicited by suprathreshold median nerve stimulation of the left hand to reach and inhibit the opposite S1 via S1-M1-M1-S1 connections. In the present study, however, the most prominent ISI for inhibiting the N20 response in the left S1 was much shorter and ranged between 20 and 25 ms, suggesting the operation of a more direct S1-S1 functional connection.

Finally, future studies might shed more light on the behavioral relevance of interhemispheric inhibition in S1 not only in healthy subjects but also in specific patient populations with altered sensory perception such as chronic stroke, multiple sclerosis or dystonia.
